# Remote Digital Health Interventions to Support the Physical, Functional, or Psychological Rehabilitation of Adult Patients With Major Traumatic Injuries: Protocol for a Systematic Review of Randomized Controlled Trials

**DOI:** 10.2196/67675

**Published:** 2025-07-28

**Authors:** Hiyam Al-Jabr, Emma Salt, John Stephenson, Esra Hamdan, Toby Helliwell

**Affiliations:** 1 Faculty of Medicine and Health Sciences Keele University Staffordshire United Kingdom; 2 Department of Research and Innovation Midlands Partnership University NHS Foundation Trust Stafford United Kingdom; 3 National Rehabilitation Centre Nottingham University Hospitals Nottingham United Kingdom; 4 School of Medicine University of Nottingham Nottingham United Kingdom; 5 Department of Allied Health, Sport and Exercise School of Human and Health Sciences University of Huddersfield Huddersfield United Kingdom; 6 Physiotherapy Department Faculty of Health Professions Al-Quds University Jerusalem Occupied Palestinian Territory

**Keywords:** digital health, mHealth, mobile health app, telemedicine, rehabilitation, physical trauma, major trauma, major injury

## Abstract

**Background:**

The use of digital health (DH) interventions has increased over the past 2 decades, providing patients with alternative remote pathways for receiving health care services. Patients with major trauma frequently require long-term access to health care services to support their mental and physical health and their overall quality of life. DH interventions can help patients stay connected to rehabilitation services, thereby enhancing their health condition and helping them regain their independence, which will enable them to return to the workplace or regain a role in society. There is a need to explore existing evidence on the effectiveness of DH interventions for improving health-related outcomes in patients with major trauma.

**Objective:**

This review aims to identify DH interventions that support the physical, functional, or psychological rehabilitation of patients who have experienced major physical trauma.

**Methods:**

This review targets randomized controlled trials. Studies investigating DH interventions in adult patients with major traumatic physical injuries (end users of the interventions) are considered eligible for inclusion. Digital interventions that are delivered remotely and studies that report the impact of DH interventions on patients’ health-related outcomes will be included. The search will be limited to publications since 2000 and peer-reviewed journals. No language restrictions will be applied, and articles not written in English will be translated. The search will be conducted in MEDLINE, Embase, AMED, CINAHL Plus, and PsycInfo. Grey literature and bibliographies of included studies and relevant reviews will also be searched for potentially relevant articles. A minimum of two reviewers will independently screen retrieved references. Data extraction will be conducted by 1 reviewer and independently checked by another reviewer. Quality assessment of the included studies will be conducted using the Cochrane Risk of Bias 2 tool. Any disagreements arising at any stage of the review will be resolved through discussion or by consulting a third reviewer, if required. A meta-analysis will be performed where possible, and a descriptive analysis of the included studies will be reported.

**Results:**

As of January 2025, the systematic review is in the data extraction stage. Seven studies have been identified as eligible for inclusion. The findings are expected to be published in a peer-reviewed journal by December 2025.

**Conclusions:**

The review findings will help identify existing evidence regarding DH interventions used to support the physical, functional, or psychological rehabilitation needs of patients with major trauma. This would help guide practitioners and policy makers to implement effective interventions to better support patient outcomes. The evidence synthesized from this review will also identify existing gaps and direct future research.

**Trial Registration:**

PROSPERO CRD42023485748; https://www.crd.york.ac.uk/PROSPERO/view/CRD42023485748

**International Registered Report Identifier (IRRID):**

DERR1-10.2196/67675

## Introduction

### Background

The last 2 decades witnessed a significant development in information technology to the point that it has become widely available in almost all aspects of modern life [1]. This has been greatly influenced by the innovative development of devices, the widespread implementation of various performing networks (eg, Microsoft Teams), and more recently, the rising need for remote delivery associated with the COVID-19 pandemic [2,3]. This has strongly contributed to driving improvements in the use of technology in health care and introducing new concepts in health care delivery through the use of digital health (DH) [1,4,5].

DH plays a significant role in health care, and it can be seen in many medical specialties [6,7]. Several definitions exist for DH [5], often with different terms used interchangeably, including DH care [1,4], telehealth [1,5,8], telemedicine [6,8-10], or telecare [11]. However, DH is the umbrella term that encompasses all other terms [12-14].

DH tools can be delivered anywhere. They can be delivered in different health care settings, with both the health care professional (HCP) and patient present in these settings, or at the patient’s own residence, where the patient can use a digital tool to track, document, and monitor their health and can have an opportunity to communicate with the HCP [6]. Thus, DH provides a wider scope of care and interventions that aim to reduce associated pressures on health care systems [8].

Traumatic injuries represent a significant cause of early death and morbidity, especially among the working population [15,16]. Globally, traumatic injuries are reported to cause around 5 to 6 million deaths each year [16,17], with around 40 million people left with permanent impairments and 100 million left with transient impairments [18-21], and there is a rising need for prolonged rehabilitation care to adjust to postinjury life. Rehabilitation is described as “a process of assessment, treatment, and management with ongoing evaluation by which the individual (and their family/carers) are supported to achieve their maximum potential for physical, cognitive, social, and psychological function, participation in society and quality of living” [22]. While there has been a recognition that more lives are being saved, rehabilitation following trauma is greatly behind, with global estimates of at least one in every 3 people needing rehabilitation services at some point throughout their injury [23]. Many people who have had major trauma are left with disability, medical dependency, family disruption, and ongoing psychosocial issues [16,24]. There is additionally a financial burden associated with supporting the rehabilitation needs of patients after major trauma [25,26].

A traumatic injury is defined as any injury that requires admission to a hospital at the time of injury [15]. According to the National Institute for Health and Care Excellence (NICE), a major trauma or injury is also defined as an injury or a combination of injuries that are life-threatening or life-changing and that could result in long-term disability [27,28]. This includes musculoskeletal injuries, traumatic brain injuries (TBIs), spinal cord injuries (SCIs), multiple fractures, and traumatic amputation [29].

Traumatic injuries have negative consequences, both physically and mentally [15]. SCIs, for example, hinder the patient’s ability to access health care, affecting their mobility and transportation [30,31] and thus disrupting the patient’s quality of life [32,33]. On the other hand, TBIs affect memory, executive functions, and cognitive skills associated with planning and decision making [34,35], leading to long-lasting activity-limiting impairment [36], disability [37,38], and changed health care needs [39]. These injuries are usually associated with psychological consequences that require equal attention [29].

Mild injuries are usually treated at home with minimal time spent in the hospital when needed [29]. However, major injuries usually require more intensive or specialized care to properly manage the patient’s condition [29]. Following traumatic injuries, patients still need rehabilitation support to help them regain their optimum function and independence [15]. However, several challenges are encountered with providing continuous services in the health care setting, including a lack of available beds in rehabilitation facilities, a dwindling number of workers in these facilities, the location of rehabilitation facilities far from a person’s home and from family members (eg, people living in rural areas), and the difficulty for people with different injuries to travel to rehabilitation facilities to receive rehabilitation support [40-43]. There is therefore a need to continue delivering services using alternative and remote pathways to keep patients connected to rehabilitation and health care services [41]. DH is a cost-effective solution that is increasingly being used to support people with different traumatic injuries [44-47].

Similar results were reported for services delivered face-to-face and those delivered in a virtual remote environment [48]. Over the past decade, the advancement and wide availability of information and communication technologies have been associated with an increase and expansion of the use of remote approaches to deliver medical and rehabilitation services [35,49,50]. Several DH interventions have been designed in the past decades to support people with different types of traumatic injuries.

### Aims and Objectives

This review aims to (1) identify DH interventions that support the physical, functional, or psychological rehabilitation of adult patients who have experienced major high-impact physical traumatic injuries and (2) assess the impact of these interventions on health-related outcomes.

The review attempts to answer the following questions:

1. What types of DH interventions are being used?
2. Which health care conditions are currently supported by DH interventions?
3. What is the impact of DH interventions on patient health-related outcomes?

## Methods

### Criteria for Considering Studies for This Review

#### Types of Studies

For this systematic review, randomized controlled trials (RCTs) with associated patient-reported health-related outcome measures will be considered eligible for inclusion. Completed studies that are published in peer-reviewed journals will be included. Other study designs, such as case reports, case studies, qualitative studies, and reviews, will be excluded. All languages will be considered, and studies that are written in a language other than English, which are eligible for inclusion, will be translated using the Online Doc Translator website [51] to confirm their eligibility. Furthermore, corresponding authors will be contacted to confirm the accuracy of the data extracted from the translated studies that will be included in the review.

#### Participants

The review will target studies that are conducted with adult patients (aged 18 years or older) who report having experienced major physical high-impact trauma or injury, who have had a period of inpatient hospitalization due to their injury, and who have received remote rehabilitation support through a DH tool. This review will focus on high-impact injuries that are usually treated in major trauma units or services (eg, TBIs, SCIs, and road traffic accidents) [52-54]. Other types of traumas will not be included as they follow a different treatment pathway to high-impact injuries (eg, fractured neck of the femur linked to fragility or injuries that can be the result of low-velocity trauma, such as a fall from standing as opposed to a fall from a high-speed motor vehicle collision). The level of severity of the traumatic injury will depend on how it is described by the included studies, which may include the injury severity score or other measures [55].

#### Interventions

Studies involving DH interventions that are used to improve physical, functional, or psychological rehabilitation and are remotely delivered or utilized by patients will be considered for inclusion. Rehabilitation interventions include interventions involving treating, assessing, managing, or evaluating individuals for the purpose of improving their health-related outcomes [22]. DH interventions that are only delivered in the health care setting (eg, hospital or clinic) will not be included in this review. Additionally, DH interventions that are directed to HCPs, students, family members, parents, or carers, and blended interventions where the impact of the targeted DH intervention on patients cannot be identified or distinguished will be excluded. No restriction will be imposed on the type of control group to be included in this review.

#### Outcomes Measured

This review will only include studies that report patient health-related outcomes associated with the use of DH interventions. This will help in identifying the impact of DH interventions on these outcomes and therefore allow drawing conclusions on their potential usefulness in supporting the care of patients with major traumatic injuries. Based on the focus of this review, reported outcomes will primarily focus on physical patient outcomes (eg, improving activity, movement, or physical function) or psychological patient outcomes (eg, improving cognition, reducing depression, or improving mental function). Studies that only report outcomes that are not related to health (eg, intervention satisfaction or acceptability, or feasibility of delivering the intervention) will not be included. The review will also mention the tools used in measuring the reported outcomes, if any.

### Search Methods for the Identification of Studies

#### Electronic Searches

A search will be conducted systematically by the main researcher using the following electronic databases: MEDLINE, Embase, AMED (via Ovid), CINAHL Plus, and PsycInfo (via Ebsco). Preliminary searches will be initially conducted using these databases to identify relevant keywords, and the final search strategy will then be developed in consultation with an information specialist with expertise in developing search strategies for systematic reviews in order to identify published relevant studies focusing on DH interventions, rehabilitation, and major trauma.

Table 1 provides the keywords that will be used to search the databases for eligible studies. The search will be limited to studies published since 2000, as this time period is associated with a wide range of technological innovations that allow patients and service users to gain easier access to the world of medicine [56-58]. An example of the full search strategy is provided in Multimedia Appendix 1.

**Table 1:** Search keywords.

Keyword heading	Keywords^a^
Digital health	telemedicine or “e?health” or “electronic health” or “m?health” or “mobile health” or “e?medicine” or e?therapy or “health technolog?” or “information technolog*” or “communication technolog*” or “mobile technolog*” or tele?care or tele?communication or tele?monitoring or “remote monitor*” or “remote consult*” or telephone or phone or smart?phone or wearable or smart?watch or internet or web?based or e?mail or “electronic mail” or online or wireless or “mobile app*” or app* or “digital health” or “digital health?care” or tele?health or “remote health*” or internet?based or computer?based or e?learning or electronic?health or electronic?learning or video?gam* or gaming or “game-based” or gamification or “Virtual Reality” or “augmented reality” or “artificial intelligence” or “Internet of Things” or technology or virtual or teletherapy or “medical technology” or “mobile application” or teleconsultation or “virtual medicine” or “video consultation” or telepsychiatry or telepsychology or telerehabilitation or tele?therapy
Rehabilitation	Rehabilitation or “Exercise Therapy” or “exercise rehabilitation” or physiotherapy or “physical therapy” or “physical rehabilitation” or “cognitive rehabilitation” or “cognitive therapy” or “psychological rehabilitation” or “psychological therapy” or “mental rehabilitation” or “mental therapy” or “musculoskeletal rehabilitation” or “physical therapy modalities” or “occupational therapy” or “post?trauma rehabilitation” or “occupational rehabilitation” or “post?traumatic rehabilitation” or “rehabilitation exercise” or “vocational rehabilitation” or kinesiotherapy or “neurologic rehabilitation” or “neurological therapy” or “recreation therapy” or “recreation rehabilitation”
Trauma or injury	“traumatic injur*” or “musculoskeletal trauma*” or “complex fracture” or fracture* or “traumatic brain injur*” or “spinal cord injur*” or “traumatic amputation” or “major trauma” or “brain injur*” or “brain trauma*” or “musculoskeletal injury” or “posttraumatic stress disorder” or “PTSD” or “acquired brain injur*” or “physical injur*” or “physical trauma*” or injur* or trauma* or “multiple trauma” or “multiple injur*” or “soft tissue injur*” or “soft tissue trauma*” or “nervous system injur*” or “nervous system trauma*” or “athletic injur*” or “athletic trauma*”

^a^The keywords of different headings are combined using “AND.”

#### Searching Other Resources

##### Reference Search

The reference lists of all studies included for final analysis and of relevant reviews will be inspected to identify previously conducted studies that might be relevant to this review.

##### Author Contact

Authors will be contacted for any missing data. Studies will not be included if there is ambiguity and if the authors cannot be contacted for clarification.

##### Grey Literature Search

A grey literature search will be conducted using the same search strategy to identify additional studies that might be useful for this review. This will be conducted using the OpenGrey website [59].

### Inclusion and Exclusion Criteria

The study inclusion criteria are as follows: (1) research that is focused on a DH intervention to support the physical, functional, or psychological rehabilitation of patients with major physical trauma; (2) primary end user of the DH intervention is an adult patient (aged 18 years or older); (3) DH intervention with potential for direct interaction with a HCP; (4) any form of digital-based intervention or treatment delivered by any digital means (eg, website or app) over any time frame; (5) intervention delivered remotely at the patient’s own residence (no need to be in an office, clinic, or health care setting); (6) research that reports patient health-related outcomes (any reported health care outcome); (7) RCT with a comparison or control group; and (8) original research (article or journal article).

Studies that do not meet one or more of the above criteria or that meet any of the following exclusion criteria will be excluded from the review: (1) DH intervention that is not focused on the physical, functional, or psychological rehabilitation of patients; (2) research that includes patients with minor or low-impact trauma or injury; (3) research focused on healthy people or public members; (4) DH intervention that is only delivered at a hospital or health care setting; (5) study where the researcher or HCP needs to perform home visits to deliver the intervention; (6) DH intervention end user is a patient carer or caregiver, family member, HCP, or student (eg, medical or nursing student); (7) research that includes caregivers or family members with reported outcomes that cannot be distinguished from patient-reported outcomes; (8) research with no reported outcomes or that only reports outcomes that are not related to health (eg, feasibility, acceptability, satisfaction, or economic evaluation); (9) research that includes mixed patient cohorts with several underlying conditions having no specific links to reported outcomes; (10) research where the underlying cause of injury is mixed (eg, traumatic and nontraumatic SCIs); (11) research that focuses on stroke or poststroke, burns, concussion, or stress after intensive care unit discharge; (12) research that focuses on psychological health rehabilitation that is not secondary to physical traumatic injury; (13) research that predicts the occurrence of an outcome or where the digital intervention is used as a screening tool; (14) digital intervention validation research; and (15) study design that is not considered an RCT (eg, quasiexperimental design, pretest/posttest cohort study, qualitative research, observational study, cross-sectional study, and review).

### Data Collection and Analysis

#### Study Selection

Search results obtained from all databases will be exported into the reference manager EndNote 9.3.3 for reference management and removal of duplicates. A double review process by 2 reviewers will be independently carried out at all screening stages to check the eligibility of all retrieved records against the inclusion criteria. Screening will be conducted using Covidence software [60]. Any arising discrepancies will be resolved by discussion between the reviewers, and a third reviewer will be consulted, if necessary. Interrater agreement will be measured using the Cohen kappa coefficient.

The search results and final findings will be presented in a PRISMA (Preferred Reporting Items for Systematic Reviews and Meta-Analyses) flowchart, including summaries of the number of studies included or removed throughout the screening process, with reasons for exclusion provided for the full-text screening.

#### Data Extraction

A data extraction template using an Excel sheet (Microsoft Corp) will be designed to extract relevant data from each eligible study, where possible. Table 2 displays the data that will be extracted from the included studies.

**Table 2:** Data to be extracted from the included studies.

Data category	Details to be extracted
General characteristics	Study title, authors, publication year, study design, and country
Characteristics of included participants	Sample size of patient participants, demographics of recruited patient participants, type of traumatic injury under investigation, and people involved in delivering the digital health intervention (eg, health care professionals)
Characteristics of the digital health intervention	Type of digital health intervention, duration and mode of intervention delivery, control group, outcomes, outcome measures, and impact or effect

Data from each eligible study will be independently extracted by 1 reviewer and checked by a second reviewer to verify the accuracy and completeness of all data extracted. Disagreements will be resolved by discussion and consensus or by consulting a third reviewer, if necessary.

#### Assessment of Risk of Bias in the Included Studies

The need for quality assessment of the identified studies will be determined once data extraction begins. Two reviewers will independently assess the risk of bias in the included studies using the Risk of Bias 2 (RoB 2) tool [61,62]. This tool assesses the risk of bias in 5 domains in RCTs: randomization process, deviations from intended interventions, missing outcome data, measurement of the outcome, and selection of the reported result. Any arising disagreement will be resolved by discussion or by consulting a third reviewer, if needed.

#### Strategy for Data Synthesis

The results of the search from all databases will be fully reported in the final document and presented in a PRISMA flowchart. A description of all included studies will be provided in tables to summarize extracted data. Study participant characteristics and intervention specifications will be presented, as reported in the original articles, to enable comparisons across studies. The quality ratings of the included studies will also be presented.

We will undertake a meta‐analysis if the participants, interventions, comparisons, and outcomes are judged to be sufficiently similar to be combined in order to arrive at an answer that is clinically meaningful. Results will be pooled from the trials using fixed effects or random effects models, considering the issue of trial methodological and clinical heterogeneity, and will be reported diagrammatically using forest plots. Where the issue of trial methodological and clinical heterogeneity appears to exist, we will also consider strategies, including not pooling data and conducting subgroup analyses or sensitivity analyses. Where data cannot be pooled due to high heterogeneity, we will still provide descriptive analysis of trial results and report them in the text of the review.

Where meta‐analyses are possible, for continuous outcomes, we will use the inverse variance method for fixed effects models and the DerSimonian and Laird variant of the inverse variance method for random effects models. For dichotomous outcomes, we will use the Mantel‐Haenszel method for fixed effects models and the DerSimonian and Laird method for random effects models. A 0.5 zero‐cell correction will be applied in the event of zero frequencies.

For studies with multiple treatment groups, we will aim to combine treatment groups to facilitate a single pairwise comparison following the methods recommended by Cochrane [63].

We will base our analyses on change scores where all necessary data, including baseline and follow-up scores and correlations, are provided; otherwise, we will use follow-up scores. Where not provided directly, we will calculate SDs from reported standard errors or CIs, or estimate from other statistics, such as IQR, or graphical representations. We will conduct sensitivity analyses to assess the influence of individual studies and represent the findings on influence plots. We will consider representing small-scale effects using funnel plots following the methods recommended by Cochrane [63] and subject to a minimum of 10 included studies.

We will use Stata statistical software (StataCorp) for all meta‐analyses and use SPSS (IBM Corp).

#### Subgroup Analysis and Investigation of Heterogeneity

We have not planned for subgroup analysis and investigation of heterogeneity.

### Ethical Considerations

No ethical approval is deemed necessary for this review, as the review will be conducted by searching available evidence that does not report any personal information about individual participants.

## Results

As of January 2025, the systematic review is in the data extraction stage. Seven studies have been identified as eligible for inclusion in this review, focusing on people with TBIs and SCIs, and describing the use of different DH remote interventions. Following the completion of data extraction and assessment of risk of bias, the findings are expected to be published in a peer-reviewed journal by December 2025. The review is registered on PROSPERO (registration number: CRD42023485748).

## Discussion

### Principal Findings

This review is designed to identify published RCTs that investigate the use of remotely delivered DH interventions and their impact on supporting the physical, functional, or psychological rehabilitation of adult patients with major physical high-impact traumatic injuries. Identifying this would add more information to current knowledge on the use of DH interventions in rehabilitation care and would establish the extent of the use of these interventions and their potential impact for improving patient outcomes.

Major traumatic injuries can drive problems with the patient’s mobility and access to health care services, and depending on the type of injury, other associated symptoms may include changes in breathing, swallowing, drinking, and cognitive functioning, and may cause depression and anxiety [15]. Major trauma thus puts patients at risk for chronic health conditions that can become life-threatening if not adequately managed [64]. Major trauma is a common cause of death in adults younger than 40 years. Various traumatic injuries demand different rehabilitation support [15]. Therefore, it is important to address the rehabilitation care needs of individual patients. Patients discharged home following acute inpatient care for their major traumatic injuries are usually still in need of continuous long-term rehabilitation support. Effective DH interventions that will be identified in this review are expected to benefit these patients by supporting their needs and improving their health-related outcomes and overall independence.

### Comparison With Prior Work

DH has undergone great development in terms of its application, growth, and reach. The number of DH interventions is increasing worldwide, as evidenced by the growing number of scientific publications, which has been greatly influenced by the COVID-19 pandemic [1]. There is additionally growing development of DH interventions for managing patients with traumatic injuries [65-68]. A wide range of DH tools have been described by previous studies in rehabilitation services, including the use of remote communication pathways such as emails, text messages, and video conferencing [69,70]. Positive outcomes were also reported by previous studies on the impact of telerehabilitation in improving patient outcomes [71,72]. Therefore, there are promising expectations for the use and impact of DH interventions in patients with major trauma.

### Strengths and Limitations

This review has several strengths. To the best of the authors’ knowledge, this is the first review to investigate the use of DH interventions in supporting people with major traumatic injuries. The findings of the review are expected to address, inform, and minimize gaps in DH rehabilitation specific to this patient population. The review will adhere to the PRISMA guidelines related to conducting systematic reviews [73] and will use a rigorous strategy, including searching related databases and checking the bibliography of the included studies and relevant reviews to identify additional relevant studies. Furthermore, no language restriction will be used in the search strategy to reduce the risk of missing studies that might be relevant to this review. Moreover, to reduce the risk of selection bias, the screening of retrieved studies and the quality assessment of included studies will be conducted by 2 reviewers, and data extraction will be checked by another reviewer, with disagreements resolved via discussion or by consulting a third reviewer, where appropriate.

The review has some limitations. The review will only focus on studies that include patients with major traumatic injuries; thus, studies with a mixed cohort of patients with various degrees of injury severity will be rejected. This may lead to missing the identification of useful interventions. However, if a distinction is made regarding the impact of the DH intervention on the various injury levels in the included cohort sample, the study will be included. Another limitation is that the review does not investigate a specific DH intervention or outcome measure. This approach might identify studies with a wide range of different DH interventions and outcomes, reducing the possibility of conducting a meta-analysis of the included studies. Upon completion of this review, further strengths and limitations might be identified and summarized.

### Future Directions

DH interventions provide an avenue to support the rehabilitation needs of patients, especially using remote pathways, when challenges exist in providing face-to-face support. The findings of this review will help identify the interventions currently available to support patients with major traumatic injuries, the types of major injuries being targeted by DH interventions, the impacts these interventions have on patient health-related outcomes, and the prioritization of resources toward rehabilitation interventions that are most effective or have the largest evidence base. Additionally, by identifying existing evidence, this review could support drawing conclusions to inform policy makers and guide HCPs to implement effective interventions in practice to better support patient outcomes.

### Dissemination Plan

The findings of this systematic review will be disseminated through various channels to share the results with the wider academic, professional, and research communities. The findings will be published in a peer-reviewed journal, and the work will be presented at relevant scientific conferences. Additionally, links to conference presentations or posters and published peer-reviewed work will be shared on professional social media platforms such as LinkedIn.

### Conclusions

The findings of this review will highlight the available evidence on DH interventions to support physical, functional, or psychological rehabilitation in patients with major trauma, and the associated impact on patient health-related outcomes. The review results will provide directions on the available interventions that could be implemented in practice. The findings will also help identify existing gaps that warrant further research and investigation.

## Appendix

Multimedia Appendix 1 Search strategy using the Ovid MEDLINE database.

Multimedia Appendix 2 PRISMA-P (Preferred Reporting Items for Systematic Review and Meta-Analysis Protocols) checklist.

## Abbreviations

DH: digital health
HCP: health care professional
PRISMA: Preferred Reporting Items for Systematic Reviews and Meta-Analyses
RCT: randomized controlled trial
SCI: spinal cord injury
TBI: traumatic brain injury

## Data Availability

Data sharing is not applicable at this stage, as no data have been generated and presented in this protocol.
