# Supplementary material for: Remote Digital Health Interventions to Support the Physical, Functional, or Psychological Rehabilitation of Adult Patients With Major Traumatic Injuries: Protocol for a Systematic Review of Randomized Controlled Trials
Source: JMIR Res Protoc. 2025 Jul 28;14:e67675. doi: 10.2196/67675 (PMC12340456; doi:10.2196/67675)
Supplement: Multimedia Appendix 1 [file resprot_v14i1e67675_app1.docx]

**Multimedia Appendix 1.** Search strategy using the Ovid MEDLINE database.

**Ovid MEDLINE(R)** ALL <1946 to December 01, 2023>

1 "Wounds and Injuries"/ 81641

2 exp Amputation, Traumatic/ 5086

3 athletic injuries/ 31158

4 exp crush injuries/ 1411

5 exp fractures, bone/ 211169

6 occupational injuries/ 3508

7 exp spinal cord injuries/ 56268

8 exp trauma, nervous system/ 239917

9 exp Multiple Trauma/ 13776

10 exp Brain Injuries/ 83599

11 Soft Tissue Injuries/ 6739

12 stress disorders, traumatic/ 756

13 exp Psychological Trauma/ 2012

14 stress disorders, post-traumatic/ 42029

15 PTSD.ti,ab,kf. 34674

16 ("posttraumatic stress disorder" or "post-traumatic stress disorder").ti,ab,kf. 40295

17 ((trauma* or wound* or crush or bone or occupational or "spinal cord" or brain or physical or mental or cognitive or multiple or "soft tissue" or "nervous system" or athletic or burn*) adj5 (injury or injuries or injured)).ti,ab,kf. 270955

18 ((musculoskeletal or amputation or wounds or crush or bone or occupational or "spinal cord" or brain or Psychological or physical or mental or cognitive or multiple or "soft tissue" or "nervous system" or athletic or burn*) adj5 (trauma or traumatic or traumas)).ti,ab,kf. 97153

19 ((bone or bones or complex or multiple) adj5 (fracture or fractures)).ti,ab,kf. 48897

20 1 or 2 or 3 or 4 or 5 or 6 or 7 or 8 or 9 or 10 or 11 or 12 or 13 or 14 or 15 or 16 or 17 or 18 or 19 779268

21 telemedicine/ 38433

22 Information Technology/ 845

23 Remote Consultation/ 5783

24 Telecommunications/ 5059

25 telephone/ 13453

26 cell phone/ 10133

27 Smartphone/ 9481

28 wearable electronic devices/ 8167

29 fitness trackers/ 1151

30 smart glasses/ 204

31 internet/ 82024

32 electronic mail/ 2992

33 satellite communications/ 1287

34 videoconferencing/ 2347

35 wireless technology/ 4718

36 Mobile Applications/ 11944

37 Video Games/ 7326

38 gamification/ 116

39 virtual reality/ 5898

40 Haptic Technology/ 222

41 augmented reality/ 1298

42 exp Artificial Intelligence/ 184148

43 Digital Technology/ 792

44 exp educational technology/ 114907

45 (telemedicine or tele-medicine).ti,ab,kf. 26111

46 (e-health or ehealth).ti,ab,kf. 11554

47 (m-health or mhealth).ti,ab,kf. 10793

48 (e-medicine or emedicine).ti,ab,kf. 103

49 (electronic adj2 (health or medicine or learning)).ti,ab,kf. 34867

50 "mobile health".ti,ab,kf. 9232

51 ((health or information or communication or mobile or haptic or digital or education* or medical or wireless*) adj3 tech*).ti,ab,kf. 101132

52 (telecare or tele-care).ti,ab,kf. 1031

53 (telecommunication or tele-communication).ti,ab,kf. 3207

54 (telemonitoring or tele-monitoring).ti,ab,kf. 2666

55 (remote* adj3 (monitor* or consult* or health*)).ti,ab,kf. 11360

56 (phone* or telephone*).ti,ab,kf. 122449

57 smartphone*.ti,ab,kf. 24879

58 (mobile* adj3 (device* or tool* or app*)).ti,ab,kf. 22090

59 (wearable adj3 (device* or electronic*)).ti,ab,kf. 12249

60 (smart-watch* or smartwatch*).ti,ab,kf. 1388

61 ((fitness* or activit*) adj2 tracker*).ti,ab,kf. 1482

62 (fitbit* or "apple watch*").ti,ab,kf. 1612

63 internet*.ti,ab,kf. 77381

64 (website* or web-site* or web-page* or webpage* or web-based).ti,ab,kf. 93262

65 (email* or e-mail* or "electronic mail*").ti,ab,kf. 24830

66 "satellite communication*".ti,ab,kf. 297

67 (videoconferenc* or video-conferenc*).ti,ab,kf. 5801

68 "digital health*".ti,ab,kf. 9019

69 ("video gam*" or videogam*).ti,ab,kf. 6128

70 (gaming or gamification).ti,ab,kf. 7148

71 virtual*.ti,ab,kf. 176900

72 "augmented reality".ti,ab,kf. 4715

73 ("artificial intelligence" or AI).ti,ab,kf. 77489

74 "video consultation*".ti,ab,kf. 872

75 (teleconsultation* or tele-consultation*).ti,ab,kf. 2291

76 (telehealth or tele-health).ti,ab,kf. 15139

77 (elearning or e-learning).ti,ab,kf. 5130

78 (app or apps or app-based).ti,ab,kf. 46134

79 online*.ti,ab,kf. 229522

80 (video-chat or "video chat").ti,ab,kf. 186

81 21 or 22 or 23 or 24 or 25 or 26 or 27 or 28 or 29 or 30 or 31 or 32 or 33 or 34 or 35 or 36 or 37 or 38 or 39 or 40 or 41 or 42 or 43 or 44 or 45 or 46 or 47 or 48 or 49 or 50 or 51 or 52 or 53 or 54 or 55 or 56 or 57 or 58 or 59 or 60 or 61 or 62 or 63 or 64 or 65 or 66 or 67 or 68 or 69 or 70 or 71 or 72 or 73 or 74 or 75 or 76 or 77 or 78 or 79 or 80 1233439

82 Rehabilitation/ 18700

83 "activities of daily living"/ 73754

84 early ambulation/ 3296

85 exp exercise therapy/ 64415

86 exp neurological rehabilitation/ 19376

87 exp occupational therapy/ 15074

88 exp rehabilitation, vocational/ 10632

89 exp Cognitive Training/ 204

90 Psychiatric Rehabilitation/ 732

91 physical therapy modalities/ 41191

92 exp exercise movement techniques/ 10487

93 Psychotherapy/ 58188

94 exp recreation therapy/ 143

95 (therapy or therapies).ti,ab,kf. 2600839

96 training.ti,ab,kf. 572676

97 rehab*.ti,ab,kf. 229097

98 exercise*.ti,ab,kf. 368596

99 physio*.ti,ab,kf. 1010107

100 kinesiotherapy.ti,ab,kf. 266

101 "Transitional Care"/ 1297

102 "transitional care".ti,ab,kf. 2332

103 rh.fs. 208790

104 82 or 83 or 84 or 85 or 86 or 87 or 88 or 89 or 90 or 91 or 92 or 93 or 94 or 95 or 96 or 97 or 98 or 99 or 100 or 101 or 102 or 103 4655962

105 81 and 104 239837

106 telerehabilitation/ 1060

107 (telerehab* or tele-rehab*).ti,ab,kf. 2445

108 (electronic adj3 therapy).ti,ab,kf. 274

109 (e-therapy or etherapy).ti,ab,kf. 514

110 (teletherapy or tele-therapy).ti,ab,kf. 2050

111 106 or 107 or 108 or 109 or 110 5515

112 105 or 111 243052

113 20 and 112 8556

114 randomized controlled trial.pt. 604235

115 controlled clinical trial.pt. 95474

116 randomi#ed.ab. 748248

117 placebo.ab. 243680

118 clinical trials as topic.sh. 201488

119 randomly.ab. 422276

120 trial.ti. 298254

121 114 or 115 or 116 or 117 or 118 or 119 or 120 1612464

122 113 and 121 1629
